# Zika Virus Prevention: U.S. Travelers’ Knowledge, Risk Perceptions, and Behavioral Intentions—A National Survey

**DOI:** 10.4269/ajtmh.17-0898

**Published:** 2018-05-07

**Authors:** Linda Squiers, James Herrington, Bridget Kelly, Carla Bann, Sylvia Becker-Dreps, Lola Stamm, Mihaela Johnson, Lauren McCormack

**Affiliations:** 1 Research Triangle Institute (RTI) International, Research Triangle Park, North Carolina;; 2 Gillings School of Global Public Health, University of North Carolina, Chapel Hill, North Carolina;; 3 School of Medicine, University of North Carolina, Chapel Hill, North Carolina

## Abstract

Limited data exist about U.S. travelers’ knowledge, risk perceptions, and behaviors related to the Zika virus (ZIKV). Using an internet research panel, in March 2017, we surveyed 1,202 Americans in the continental United States and Puerto Rico who planned to travel to a ZIKV-affected country, state, or U.S. territory in 2017. We compared levels of knowledge and perceived risk of ZIKV, and intentions to practice ZIKV prevention behaviors across respondents from three regions: Puerto Rico, at-risk states, and other states. More than 80% of respondents correctly understood that a person could acquire ZIKV through a bite from an infected mosquito, and over 64% of respondents knew that a pregnant woman could pass the virus to her fetus. Less than half of the respondents from at-risk states and other states knew that ZIKV could be transmitted sexually, as compared with three-quarters of respondents from Puerto Rico. Compared with respondents from at-risk and other states, respondents from Puerto Rico were the most knowledgeable for almost all types of knowledge assessed. Knowledge about post-travel precautions was low across all three regions. Differences in perceived risk and intentions to practice specific prevention behaviors also varied among regions. Significant gaps exist in U.S. travelers’ knowledge about how to prevent ZIKV transmission both during and after travel. Input and collaboration from the travel industry, health care providers, and the media are needed to help educate travelers about how to prevent ZIKV infection and transmission.

## INTRODUCTION

The Zika virus (ZIKV) is unique among the more than 100 viruses that can cause encephalitis, meningitis, and hemorrhagic disease in humans, in that ZIKV can be transmitted through the bite of female mosquito vectors *Aedes aegypti* and *Aedes albopictus*; through sexual contact between humans; congenitally, from a pregnant woman to her fetus; and through blood transfusion.^1,2^ ZIKV prevalence and incidence were essentially unknown in the Western Hemisphere until early 2015 when reports were observed in Brazil of associations between ZIKV infection and individuals presenting with Guillain–Barré syndrome, and mothers delivering children born with microcephaly and other congenital malformations.^3,4^ It is now known that 8–15% of live-born infants present with these congenital defects among U.S. women who have laboratory-confirmed ZIKV infection in their first trimester, as reported to the U.S. ZIKV Pregnancy Registry.^5–7^

At present, 48 countries and territories in the Western Hemisphere have confirmed autochthonous, vector-borne transmission of ZIKV disease, and five countries have reported sexually transmitted ZIKV cases.^8^ Although only limited pockets of autochthonous transmission have been documented in the southern United States, there is the potential for future vector-borne spread, as the *Ae. aegypti* and *Ae. albopictus* competent vectors are present in multiple regions of the United States.^9,10^ Over 221,000 locally acquired cases have been confirmed in the Americas.^8^ Within the United States and its territories, ZIKV has most profoundly affected the U.S. territories, especially Puerto Rico, which had more than 35,000 locally acquired (i.e., when a person has not traveled to another area) symptomatic cases reported.^8^ In the continental United States, almost every state has reported cases of ZIKV, with most cases acquired while traveling to a ZIKV-affected area.^2^

Each year, more than 30 million U.S. residents journey south of the U.S. continental Gulf for business (20%) or leisure (80%), traveling to Mexico (71%), the Caribbean (18%), Central America (7%), or South America (5%). Peak months for U.S. residents to travel to the Caribbean are March, June, and July, which typically coincide with spring break (809,877 in March) and summer holidays (803,919 in June and 890,781 in July).^11^

Those who travel to ZIKV-affected areas are at risk of acquiring ZIKV through mosquito bites. However, mosquito bites are not the only transmission route. ZIKV is unique because it also can be transmitted sexually from a person who has acquired the virus while traveling to an area of high ZIKV prevalence to his or her partners after returning home, even if the infected person does not demonstrate symptoms at the time.^12^ Notably, ZIKV can be found in men’s semen for up to 6 months and in vaginal secretions for 1 month.^13,14^ Consequently, travelers to ZIKV-affected areas could be at risk for acquiring the virus through mosquito bites and/or sexual contact.

To our knowledge, only a few studies have reported individuals’ knowledge, risk perceptions, or behaviors regarding mosquito-borne infections, including a national poll of 1,004 adults conducted in March and August 2016.^15–18^ The most recent study found that 4 in 10 Americans had heard only a little or nothing about ZIKV. Of those who had heard of ZIKV, 90% knew that it could be spread through the bite of a mosquito carrying the virus; however, only 58% knew that it could be transmitted sexually.^18^ To date, only one study has assessed the impact of ZIKV on travel planning.^19^ Consequently, given the large number of U.S. residents that typically travel to areas that have had local transmission of ZIKV, we surveyed U.S. travelers to assess if they have the knowledge needed to protect themselves from ZIKV, whether they perceive that they are at risk for getting ZIKV, and if they intend to practice recommended ZIKV prevention behaviors.

At the end of 2016—3 months before the launch of our survey—Puerto Rico reported 34,963 cases of symptomatic ZIKV disease, with 99.6% occurring through presumed mosquito-borne infection; whereas U.S. states reported 5,102 cases, 95% of which occurred in travelers returning from ZIKV-affected areas. Given the large number of cases and high risk for ZIKV infection in Puerto Rico, the campaign *This is How We Stop Zika (Detén el Zika)*,^20^ funded through the CDC Foundation, was launched in Summer 2016 in Puerto Rico to increase awareness of the risk of ZIKV, how it is transmitted, and how to prevent it.^21^ Paid advertising for this large, multi-media campaign continued through February 2017 and resulted in over 38 million total ad impressions via traditional media, such as television, radio and newspaper ads, billboards, and social media, giving most of the 3.4 million Puerto Ricans multiple opportunities to be exposed to the campaign messages.^22^

In addition, the CDC Foundation launched the Zika Contraception Access Network (Z-CAN).^23^ Because contraceptive access in Puerto Rico was limited, this program provided services, including reversible contraceptive methods, to more than 21,000 women between May 2016 and August 2017. The Z-CAN program included a health communication campaign—called *When in Doubt, Ask (Ante la Duda, Pregunta)*—to increase women’s awareness of the program.^20,24^

Furthermore, the National Association of Chain Drug Stores Foundation sponsored a radio and online Zika prevention campaign. This effort encouraged women to speak to physicians and pharmacists about how to protect themselves and their unborn children from ZIKV.^25^

During the same period, the US Centers for Disease Control and Prevention (CDC) launched a campaign for the continental United States that had messaging for travelers, including ads placed in United Airlines and American Airlines in-flight magazines. Ads ran in both 2016 and 2017.

In response to the ZIKV cases in both Miami, FL, and Brownsville, TX, some states developed public awareness campaigns—including Florida’s “*Fight the Bite*”, Georgia’s “*Tip and Toss*”, and Texas’s “*Don’t Give Zika a Biting Chance*”^26,27^ campaigns—to help residents and travelers to these areas understand how to protect themselves and their communities from ZIKV. Because Florida, Georgia, and Texas all have major news media markets, individuals living in nearby states also had the potential to be exposed to more news coverage about ZIKV, given the local ZIKV transmission in Florida and Texas. Florida, Georgia, and Texas also have some of America’s busiest interstate highways, making it possible for travelers between these states to be exposed to additional information about ZIKV on highway billboards, as compared with other parts of the continental United States.^28^

Given the different levels of local ZIKV transmission and public education campaigns, this study sought to determine whether levels of knowledge, perceived risk, and intentions to practice ZIKV prevention behaviors varied among people living in Puerto Rico, states affected by or at-risk for ZIKV, and the remaining states in the continental United Sttates.

## MATERIALS AND METHODS

To conduct this study, we surveyed residents of the continental United States and Puerto Rico who planned to travel to a ZIKV-affected area in 2017. The survey was available in both English and Spanish.

### Data source.

We recruited respondents in the continental United States through an internet panel maintained by GfK Custom Research, LLC. The GfK KnowledgePanel^®^ (New York, NY) consists of 50,000 adult panel members recruited using address-based sampling. The GfK panel is based on probability sampling covering both the online and offline populations in the continental United States. For households that do not have a computer or internet access, GfK provides them with both so they can participate. The resulting sample includes representation from listed and unlisted telephone numbers, telephone and non-telephone households, mobile phone–only households, and households with and without internet access. Panelists are asked to respond to several surveys a month and can enter special raffles or they can be entered in special sweepstakes with both cash rewards and other prizes. For the continental U.S. states’ respondents, a random sample of 8,732 panel members was drawn from GfK’s KnowledgePanel^®^. To supplement this sample, we added an opt-in panel (not a randomized household sample) that could deliver a sample of residents in Puerto Rico via digital marketing.

### Eligibility criteria.

Eligible respondents were U.S. residents of reproductive age (aged 18–44) who had ever seen or heard something about ZIKV, and had plans to travel to a ZIKV-affected country, a ZIKV-affected U.S. territory, or an “at-risk” state (hereafter called at-risk states) in 2017. Table 1 presents definitions for each area.

**Table 1:** Definitions of ZIKV-affected destinations

ZIKV-affected and at-risk states
States included Texas and Florida, which had reported local transmission of Zika virus (ZIKV) through mosquito bites at the time the survey was fielded. We also included Alabama, Georgia, Louisiana, Mississippi, and South Carolina in this region, given the potential for ZIKV spread caused by the presence of *Aedes aegypti* mosquitoes and their likelihood to live and reproduce,^41^ the number of cases reported in the state in 2016,^36^ and their proximity to Florida and Texas.^42–44^

ZIKV-affected countries
Countries reporting local transmission of ZIKV in March 2017 included all Caribbean Islands; the Pacific Islands of Fiji, the Marshall Islands, Micronesia, New Caledonia, Palau, Papua New Guinea, Samoa, Tonga; the Central or South American countries of Argentina, Belize, Bolivia, Brazil, Columbia, Costa Rica, Ecuador, El Salvador, French Guiana, Guatemala, Guyana, Honduras, Nicaragua or Panama, Paraguay, Peru, Suriname or Venezuela; and the Southeast Asian or Asian countries of Brunei, Burma (Myanmar), Cambodia, Indonesia, Laos, Malaysia, the Maldives, the Philippines, Singapore, Thailand, Timor-Leste (East Timor), and Vietnam.^36^

ZIKV-affected U.S. territories
The U.S. territories that had reported autochthonous transmission of ZIKV in March 2017 were American Samoa, Puerto Rico, and the U.S. Virgin Islands.^36^

Given the goals of the study, we sampled 501 respondents from at-risk states, 500 respondents from all other states in the continental United States (hereafter called “other states”), and 201 respondents from Puerto Rico. All respondents were required to complete the online informed consent. The study protocol and survey instrument were reviewed and approved by both the Research Triangle Institute (RTI) International and the University of North Carolina at Chapel Hill Institutional Review Boards.

### Data collection.

After completing a brief screening survey, eligible respondents were sent a link to the online survey, which was available in English and Spanish. The survey was conducted from March 17 through April 4, 2017.

### Weighting.

Using the American Community Survey (ACS) 2015 data as benchmarks, all screened U.S. state respondents were weighted to represent U.S. residents aged 18–44, with finer demographic adjustments within the two areas (i.e., the at-risk states and the other states) by two education groups (less than high school or high school education and some college or above). We applied weights to adjust for deviations between the respondents and population distributions. Respondents were weighted to represent the general population using demographics from the ACS 2015. Demographic and other characteristics used in the calculation of weights included gender, age, race/ethnicity, Census region, metropolitan status (metro, nonmetro), education, household income, and language proficiency. The sample from Puerto Rico was weighted using the same methods but was based on a more limited set of characteristics: gender by age, household income, race/ethnicity, and education.

### Measures.

We developed survey-based measures for this study to assess travel plans, ZIKV-related knowledge, perceived risk, ZIKV-related information seeking, self-efficacy in preventing ZIKV, perceived effectiveness of ZIKV preventive methods, intentions to engage in ZIKV protective behaviors, and demographic characteristics.

#### Travel logistics.

We asked respondents to indicate the month their next trip would take place and the purpose of their travel, such as business, leisure, vacation or adventure, visiting friends or relatives, providing or receiving medical care, research or education, and mission or nonmedical service. Respondents were asked to reference their next trip when responding to all survey questions.

#### ZIKV-related knowledge.

Respondents answered eight knowledge-related questions. First, to assess respondents’ self-reported knowledge, we asked them “In general, how much would you say you know about ZIKV?” Response options ranged from 1 = not at all to 5 = a lot. Second, to assess respondents’ objective knowledge, using a “check all that apply” format, we showed them a list of symptoms and asked them to select those that were caused by ZIKV. Using the same question format, we also asked respondents to select the ways through which someone can acquire ZIKV. Third, we asked respondents to review a set of statements and select those that they believed to be true. Topics included the possible impact of ZIKV on a fetus, testing recommendations for pregnant women, sexual transmission during pregnancy, and whether *N*,*N*-diethyl-*meta*-toluamide (DEET), an insect repellant, is safe for use by pregnant women. Finally, using true or false questions, we asked respondents to indicate the length of time (in months) males needed to use condoms after exposure to ZIKV, and the length of time (in months) women needed to wait before trying to get pregnant.

#### Perceived risk.

We used three survey items to measure respondents’ perceptions of risk for becoming infected with ZIKV. We asked respondents “How concerned are you that you will get infected with ZIKV while traveling?” (measured on a 3-point scale: not at all concerned, somewhat concerned, and very concerned); “How likely is it that you will become infected with the ZIKV by having sex with someone who has ZIKV and doesn’t know it?”; and “At your travel destination, how likely is it that you will be bitten by a mosquito that is infected with ZIKV?” (the latter two questions measured on a 4-point scale from 1 = not at all likely to 4 = very likely).

#### Self-efficacy in prevention of ZIKV.

We asked respondents “How confident are you in your ability to protect yourself from ZIKV?” Response options included not at all confident, somewhat confident, confident, and very confident.

#### ZIKV information seeking.

Using yes or no questions, we asked respondents if they had actively searched for information about ZIKV online in the previous 6 months before deciding to travel, if they tried to find out whether their travel destination was affected by ZIKV, and if information about the risk of getting ZIKV affected their travel plans.

#### Intentions to use specific ZIKV prevention measures.

We listed nine ZIKV-prevention methods and asked respondents to indicate “Realistically, how likely is it that you will do the following to prevent Zika?” A 5-point Likert scale was used to capture responses, with 1 = not at all likely and 5 = very likely. Prevention methods included measures that can effectively deter mosquito bites (e.g., using a mosquito repellent; wearing long-sleeve shirts and long pants; staying in places with air conditioning, window screens, and door screens to keep mosquitoes outside; treating clothing with permethrin spray (a mosquito insecticide); sleeping under a mosquito/bed net; or reducing the risk of sexual transmission (e.g., by staying sober to avoid casual sex without a condom with someone who is in or has recently traveled to an area with ZIKV); abstaining from any type of sex with someone who is in or has recently traveled to an area with ZIKV; using a condom with someone who is in or has recently traveled to an area with ZIKV.

#### Current behavior.

We asked all respondents “Are you and your partner using a condom to prevent the possible transmission of the ZIKV?” and “Have you talked with a doctor, nurse, or other medical professional about the ZIKV?”

#### Sociodemographic characteristics.

When joining the internet panel, GfK asked respondents to indicate their age (in years), gender (male/female), race (white, black/African American, American Indian or Alaska Native, Asian, Hawaiian/Pacific Islander), ethnicity (Hispanic or non-Hispanic), education (less than high school, high school, some college, bachelor’s degree or higher), college student status (full-time or part-time), employment status (paid employee; self-employed; unemployed), marital status (married, separated, divorced, never married, living with partner), and income (less than $5,000 up to $250,000 or more).

### Analysis groups.

The study sample comprised respondents from three different regions that we believed had been exposed to different levels of ZIKV prevention messages based on local transmission rates, the presence of *Ae. aegypti* and *Ae. albopictus* mosquitoes, known ZIKV prevention campaigns, and projected local media coverage about ZIKV. The *at-risk states* study group included respondents from Alabama, Florida, Louisiana, Mississippi, and Texas, plus two states in the Southeast—Georgia and South Carolina. The *other states* study group included respondents from all other states in the continental United States. The *Puerto Rico* study group comprised respondents residing in the Commonwealth of Puerto Rico.

### Analysis.

Responses to knowledge items were dichotomized into correct and incorrect, with “don’t know” coded as incorrect. Descriptive statistics were calculated to summarize differences across the three regions based on demographic characteristics and survey responses, with frequencies and percentages for categorical variables and means and standard deviations for continuous scores. Demographic characteristics were used in the calculation of the survey weights; therefore, regional differences were assessed using weighted χ^2^ or comparison of means tests. Comparisons for the survey items and scale scores were conducted using weighted χ^2^ tests using SAS PROC SURVEYFREQ. All analyses were conducted using SAS version 9.4 (Cary, NC).

## RESULTS

For the two groups within the continental United States, a random sample of 8,075 panel members was drawn from GfK’s KnowledgePanel^®^. We calculated response rates based on standard formulas for online panel response rates.^29^ Excluding breakoffs (respondents who clicked the link to start the survey, but never finished, *N* = 97), 3,869 panel members responded to the invitation and 1,001 qualified for the survey, yielding a final stage completion rate of 48% and a qualification rate of 26%. The recruitment rate for this study, reported by GfK, was 12% and the profile rate was 63%, for a cumulative response rate of 4%.^21^ It is important to note that response rates to online panel surveys are often lower than response rates to surveys using other data collection methods, such as postal questionnaires or face-to-face interviews.^30,31^ One meta-analysis found that response rates to online surveys are, on average, 11% lower than response rates to other types of surveys.^32^ These rates can appear even lower because of the need to multiply the recruitment rate, profile rate, cooperation rate, and panel retention rate for a cumulative response rate.^29–31^

The Puerto Rico sample came from an opt-in panel where 657 began the screener and 201 qualified for the survey, for a completion rate of 31%. With opt-in panels, the probabilities of selection are unknown; consequently, it is not possible to calculate a response rate. A total of 1,202 respondents completed the survey, of whom 201 were from Puerto Rico, 501 were from at-risk states, and 500 were from other states. Prior to weighting, we found significant differences in demographic characteristics among the three regions. Once weights were applied, these differences were no longer significant for all demographic variables except income, as respondents from Puerto Rico had significantly lower levels of income than respondents from the at-risk states and the other states, as would be expected given the large difference in median income ($57,320 continental United States as compared with $19,350 Puerto Rico in 2015) and unemployment (6% continental United States as compared with 19% Puerto Rico in 2015) that exists between Puerto Rico and the continental United States.^33–35^

All respondents from Puerto Rico identified themselves as Hispanic, as compared with 27% of respondents from at-risk states and 21% from other states (Table 2). Among the respondents from Puerto Rico, 95% completed the survey in Spanish, as compared with 10% of respondents from Gulf states and 7% of respondents from non-Gulf states (χ^2^ = 760.65, *P* < 0.001). White respondents accounted for 48% of respondents from at-risk states and 55% or respondents from other states (*P* < 0.01).

**Table 2:** Respondents’ demographic characteristics (*N* = 1,202)

	At-risk states (*N* = 501)		Other states (*N* = 500)		Puerto Rico (*N* = 201)		*P* value
	*n*	%	*n*	%	*n*	%	
Age							
18–29	192	38.3	224	44.9	73	36.2	0.019
30–44	309	61.7	276	55.1	128	63.8	
Gender							
Male	221	44.0	230	45.9	84	41.9	0.034
Female	280	56.0	270	54.1	117	58.1	
Race/ethnicity							
White, non-hispanic	242	48.4	277	55.4	0	0	< 0.001
Black, non-hispanic	87	17.4	69	13.7	0	0	
Hispanic	134	26.8	106	21.1	201	100	
Other/multiple race	37	8.2	49	9.7	0	0	
Education							
Less than high school	59	11.8	43	8.5	7	3.7	< 0.001
High school	97	19.4	95	19.0	43	21.3	
Some college	186	37.1	154	30.7	69	34.3	
Bachelor’s degree or higher	159	31.7	209	41.8	82	40.7	
College student (full- or part-time)	105	21.0	114	22.7	40	20.2	0.816
Employment status							
Employed	393	78.5	397	79.4	174	86.4	0.915
Unemployed	108	21.5	103	20.6	27	13.6	
Income							
Less than $25,000	58	11.5	45	9.0	103	51.1	< 0.001
$25,000–$49,999	107	21.4	70	14.0	63	31.2	
$50,000–$74,999	101	20.1	92	18.4	27	13.4	
$75,000–$99,999	69	13.8	89	17.8	4	1.9	
$100,000 or more	167	33.3	204	40.9	5	2.5	
Marital status							0.567
Married/living with partner	314	62.6	304	60.8	133	66.2	
Not married	187	37.4	196	39.2	68	33.8	

### Travel intentions.

Significantly more respondents from at-risk states (49%) and other states (45%) planned to travel in the spring as compared with respondents from Puerto Rico (18%) (*P* < 0.001) (Table 3). Across all three regions, the most frequently reported reason for traveling was for leisure, vacation or adventure, followed by visiting friends or relatives.

**Table 3:** Respondents’ travel plans to ZIKV-affected areas (*N* = 1,202)

	At-risk states (*N* = 501)		Other states (*N* = 500)		Puerto Rico (*N* = 201)		*P* value
	*n*	%	*n*	%	*n*	%	
Timing of travel in 2017							
Spring (March–May)	246	49.4	220	44.6	37	18.4	< 0.001
Summer (June–August)	189	38.1	196	39.7	85	42.3	
Fall/Winter (September–December)	62	12.5	77	15.7	79	39.3	
Type of travel							
Business	53	10.5	45	9.0	9	4.5	0.187
Leisure, vacation, or adventure	338	67.5	346	69.3	135	67.2	0.869
Visiting friends or relatives	183	36.6	176	35.3	80	39.7	0.689
Providing or receiving medical care	6	1.1	8	1.6	2	0.9	0.739
Research or education	5	0.9	11	2.2	8	3.7	0.040
Mission or nonmedical service	6	1.2	3	0.6	1	0.6	0.561

### Knowledge.

Both perceived and actual knowledge were significantly different among the three study groups (Table 4). Respondents from Puerto Rico were significantly more likely to report knowing “5–a lot” about ZIKV (12%), as compared with respondents from at-risk states (7%) and other states (2%) (*P* < 0.001). For all items, respondents from Puerto Rico had significantly higher mean knowledge scores (64% correct) as compared with respondents from at-risk states (52% correct) and other states (52% correct) (*P* < 0.001).

**Table 4:** Respondents’ ZIKV-related knowledge: symptoms, transmission methods, and pregnancy risks (*N* = 1,202)

	At-risk states (*N* = 501)		Other states (*N* = 500)		Puerto Rico (*N* = 201)		*P* value
	*n*	%	*n*	%	*n*	%	
In general, how much would you say you know about ZIKV?							
1–nothing at all	37	8.0	33	6.5	8	7.6	< 0.001
2	161	34.3	182	36.5	23	11.3	
3	212	38.1	203	40.7	74	39.5	
4	62	12.8	70	14.4	61	30.3	
5–a lot	28	6.8	13	1.8	31	11.6	
To the best of your knowledge, which of the following ways can someone get the ZIKV? (displaying % correct)							
Receiving a transfusion with blood that contains the ZIKV (*true*)	210	42.0	227	45.3	56	27.9	0.002
Breathing the same air as a person who is sick from ZIKV (*false*)	461	91.9	472	94.4	191	94.8	0.463
Being bitten by a mosquito that carries the ZIKV (*true*)	425	84.9	433	86.6	168	83.7	0.707
Having sex with someone who is infected with the ZIKV (*true*)	210	41.9	228	45.5	155	77.0	< 0.001
During pregnancy, ZIKV can be passed from a pregnant woman to her fetus (*true*)	327	65.3	322	64.4	136	67.7	0.801
Drinking unclean water (*false*)	446	89.0	459	91.7	189	94.1	0.238
Don’t know	50	9.8	49	10.6	6	5.0	0.061
How many people infected with the ZIKV show symptoms? (% correct - “some have symptoms”)	185	36.9	202	40.6	59	29.2	0.079
Which of the following are common symptoms of ZIKV (displaying % correct)							
Mild fever (*true*)	293	59.1	264	53.1	167	83.4	< 0.001
Bloody cough (*false*)	295	59.5	278	56.0	175	87.4	< 0.001
Rash (true)	126	25.4	100	20.1	96	48.2	< 0.001
Joint or muscle pain (*true*)	225	45.4	215	43.2	154	77.2	< 0.001
Confusion (*false*)	267	53.9	273	55.0	181	90.7	< 0.001
Pink eye (*true*)	38	7.6	32	6.5	50	25.1	< 0.001
Headache (*true*)	199	40.0	175	35.1	165	82.5	< 0.001
Diarrhea (*false*)	146	29.5	139	27.9	110	54.9	< 0.001
Don’t know	170	33.9	181	36.2	9	4.5	< 0.001
Which of the following is a true statement? (displaying % correct)							
The ZIKV can cause brain or birth defects in fetuses (*true*)	403	79.8	412	81.4	173	86.1	0.330
If a pregnant woman becomes infected with ZIKV, her baby will always be infected (*false*)	316	60.1	315	61.1	131	66.5	0.463
Women infected with ZIKV during pregnancy are more likely to have a baby with birth defects than women who have not been infected with ZIKV (*true*)	378	74.4	373	74.1	167	81.1	0.309
ZIKV cannot be transmitted by sex if a woman is pregnant (*false*)	413	83.4	402	79.8	179	92.0	0.008
Pregnant women who have visited a ZIKV infected area should be tested for ZIKV whether they have symptoms (*true*)	323	64.4	297	59.9	104	52.2	0.064
Public health officials say that insecticides containing DEET are safe for pregnant women to use (*true*)	86	15.7	64	10.8	39	17.0	0.069
The ZIKV frequently causes diarrhea in adults (*false*)	336	65.0	365	71.9	167	81.6	0.002
Within the United States, there have only been cases of ZIKV in Florida (*false*)	345	69.9	346	70.9	175	86.7	0.001
Men who may have become infected with ZIKV should use a condom for at least _____ after being infected even if they have no symptoms. (displaying % correct–6 months)	105	19.1	102	18.7	37	14.7	0.448
Women who may have been exposed to ZIKV should wait _____ before trying to get pregnant. (displaying % correct–2 months)	8	2.5	9	1.8	5	2.3	0.883
Mean knowledge index score (percent correct)	261	52.1	258	51.6	129	64.0	< 0.001

DEET = *N*,*N*-diethyl-*meta*-toluamide; ZIKV = Zika virus.

When asked about potential transmission methods for ZIKV, 84–87% of respondents across the three regions correctly identified “being bitten by ZIKV infected mosquito” and more than 89% of respondents in all three regions correctly identified that ZIKV cannot be transmitted by breathing the same air as a person who is sick with ZIKV, or by drinking unclean water. More than 64% of respondents across the three regions also knew that ZIKV can be transmitted from a pregnant woman to her fetus and, notably, significantly more respondents from Puerto Rico knew that ZIKV can be transmitted by having sex with someone who is infected with ZIKV (77%) as compared with respondents from the at-risk states (42%) and the other states (46%) (*P* < 0.001). Although respondents from Puerto Rico were the most knowledgeable about sexual transmission of ZIKV, significantly more respondents from the at-risk states (42%) and the other states (45%) knew that ZIKV can be transmitted through a blood transfusion (28%) (*P* = 0.002).

A larger proportion of respondents from Puerto Rico were knowledgeable about ZIKV-related symptoms and which symptoms were not related to ZIKV, as compared with respondents from the at-risk states and the other states (*P* < 0.001) (Table 4).

Lastly, respondents in all three regions were equally knowledgeable about the impact of ZIKV on pregnancy, including that ZIKV can cause brain and birth defects in fetuses (80% or more) and that women infected with ZIKV were more likely to have a baby with birth defects than women not infected with ZIKV (over 74%). Respondents were less knowledgeable about testing for ZIKV and precautions around conception. Namely, only 52–64% of respondents knew that pregnant women should be tested for ZIKV, regardless of having symptoms, if they visited a ZIKV-affected area. Between 15% and 19% of respondents knew that men who may have become infected with ZIKV should use a condom for at least 6 months after being infected even if they had no symptoms, and 3% or fewer respondents knew that women who may have been exposed to ZIKV should wait 2 months before trying to get pregnant.

### Information seeking, perceived risk, and self-efficacy.

A significantly higher percentage of respondents from Puerto Rico (51%) reported actively looking for information about ZIKV in the past 6 months, as compared with respondents from the at-risk states (41%) and the other states (18%) (*P* < 0.001) (Table 5). Respondents from Puerto Rico (49%) were significantly more likely to report talking with a medical professional about ZIKV as compared with respondents from the at-risk states (11%) and the other states (9%) (*P* < 0.001). In the context of making travel plans, a significantly higher percentage of respondents from Puerto Rico (50%) reported trying to find out whether their travel destination was affected by ZIKV, as compared with respondents from the at-risk states (12%) and the other states (18%) (*P* < 0.001). Most respondents indicated that the risk of ZIKV had not affected their travel plans.

**Table 5:** Respondents’ information seeking, perceived risk, and self-efficacy related to ZIKV (*N* = 1,202)

	At-risk states (*N* = 501)		Other states (*N* = 500)		Puerto Rico (*N* = 201)		*P* value
	*n*	%	*n*	%	*n*	%	
Information seeking							
In the last 6 months, have you actively looked for information about ZIKV online? (% yes)	94	40.5	93	17.8	109	50.7	< 0.001
Before deciding to travel, did you try to find out whether your destination was affected by ZIKV? (% yes)	70	12.2	90	17.6	108	50.4	< 0.001
Have you talked with a doctor, nurse or other medical professional about ZIKV? (% yes)	62	10.5	48	9.2	100	49.1	< 0.001
How has information about the risk of getting the ZIKV affected your travel plans? (% yes)							
It did not affect my plans	465	92.9	448	90.8	170	89.9	0.058
I changed my travel destination	12	2.7	15	3.0	2	0.2	
I changed my travel dates	2	0.7	7	0.8	7	2.2	
I designed my original travel plans to avoid any areas with ZIKV	17	3.3	25	4.4	20	7.6	
Other	3	0.5	5.1	1.0	1	0	
Concern							
How concerned are you that you will get infected with the ZIKV while traveling?							
Not at all concerned	353	72.7	344	69.5	41	23.3	< 0.001
Somewhat concerned	123	21.7	125	26.2	86	44.8	
Very concerned	24	5.7	29	4.4	74	32.0	
Perceived risk							
How likely is it that you will become infected with the ZIKV by having sex with someone who has ZIKV and doesn’t know it?							
Not at all likely	323	65.7	315	62.9	47	29.3	< 0.001
Somewhat likely	96	20.2	90	19.2	32	12.2	
Likely	44	7.5	55	10.5	55	27.5	
Very likely	34	6.6	37	7.3	67	31.0	
At your travel destination, how likely is it that you will be bitten by a mosquito that is infected with the ZIKV?							
Not at all likely	265	49.9	246	54.2	27	12.8	< 0.001
Somewhat likely	190	42.2	207	37.0	81	37.1	
Likely	25	5.9	29	6.6	68	38.6	
Very likely	13	1.9	9	2.2	23	11.6	
Self-efficacy							
How confident are you in your ability to protect yourself from ZIKV?							
Not at all confident	59	11	66	13.8	8	4.0	< 0.001
Somewhat confident	240	46.3	259	52.2	59	31.7	
Confident	119	24.7	122	24.7	105	52.6	
Very confident	80	18.1	47	9.2	29	11.7	
Current condom use							
Are you and your partner using a condom to prevent the possible transmission of the ZIKV? (% yes)	68	16.6	78	19.5	62	31.5	< 0.004

ZIKV = Zika virus.

Respondents from Puerto Rico (77%) were significantly more likely to report feeling somewhat or very concerned that they would get infected with ZIKV while traveling, as compared with respondents from the at-risk states (27%) and the other states (31%) (*P* < 0.001). Respondents from Puerto Rico (59%) also were significantly more likely to report they were likely or very likely to become infected with ZIKV by having sex with someone who has ZIKV and does not know it, as compared with respondents from the at-risk states (14%) and the other states (18%) (*P* < 0.001). Similarly, more respondents from Puerto Rico (50%) reported being likely or very likely to get ZIKV through a bite from an infected mosquito at their travel destination, as compared with respondents from the at-risk states (8%) or the other states (9%) (*P* < 0.001). We found significant differences among respondents in the three regions with respect to confidence in their ability to protect themselves from ZIKV. Significantly more respondents from Puerto Rico (64%) indicated they were confident or very confident as compared with respondents from the at-risk states (43%) and the other states (34%) (*P* < 0.001).

### Behavior and behavioral intentions.

Respondents from Puerto Rico (35%) were significantly more likely to say they would be very likely to wear long sleeve shirts and long pants to prevent mosquito bites, as compared with respondents from the at-risk states (25%) and the other states (15%) (*P* < 0.001) (Table 6). However, among the three regions, significantly fewer respondents from Puerto Rico (28%) said they would be very likely to stay in places with air conditioning and screened doors and windows, as compared with respondents from the at-risk states (47%) and the other states (35%) (*P* = 0.003). Overall, sleeping under a bed net was not a behavior that respondents intended to do. Only 21% of respondents from Puerto Rico, 17% of respondents from the at-risk states, and 12% of respondents from the other states indicated they were very likely to sleep under a bed net (*P* = 0.034).

**Table 6:** Respondents’ likelihood of practicing different ZIKV prevention behaviors (*N* = 1,202)

		At-risk states (*N* = 501)		Other states (*N* = 500)		Puerto Rico (*N* = 201)		
Item	Value	*n*	%	*n*	%	*n*	%	*P* value
Realistically how likely is it that you will do the following to prevent ZIKV…								
Use mosquito repellant	Not at all likely	48	9.8	52	10.0	19	10.7	0.115
	Somewhat likely	85	18.1	103	21.0	28	16.3	
	Likely	147	28.8	155	32.5	47	20.9	
	Very likely	216	43.2	179	36.5	102	52.2	
Wear long sleeve shirts and long pants	Not at all likely	128	25.7	132	27.7	22	11.7	< 0.001
	Somewhat likely	136	26.8	158	32.5	44	24.4	
	Likely	128	22.9	120	25.0	61	29.2	
	Very likely	102	24.6	81	14.9	69	34.8	
Stay in places with air conditioning window screens and door screens to keep mosquitoes outside	Not at all likely	47	8.7	60	11.8	28	17.3	0.003
	Somewhat likely	83	16.0	102	21.1	42	23.8	
	Likely	146	28.7	159	32.3	56	31.2	
	Very likely	219	46.6	170	34.8	69	27.7	
Treat clothing with permethrin spray (an insecticide that kills mosquitoes)	Not at all likely	197	38.2	186	37.3	58	36.2	0.206
	Somewhat likely	129	24.9	132	28.0	42	18.9	
	Likely	91	18.5	102	21.1	53	27.3	
	Very likely	77	18.5	70	13.6	41	17.6	
Sleep under a mosquito/bed net	Not at all likely	255	51.8	240	49.5	61	36.7	0.034
	Somewhat likely	85	18.5	97	21.4	41	23.2	
	Likely	76	13.0	96	17.3	43	18.7	
	Very likely	77	16.7	56	11.9	52	21.4	
Stay sober to avoid casual sex without a condom with someone who is in or has recently traveled to an area with ZIKV	Not at all likely	125	26.1	127	25.3	38	18.8	0.01
	Somewhat likely	57	11.1	75	16.9	36	22.5	
	Likely	78	14.8	88	17.5	42	23.2	
	Very likely	233	48.0	198	40.4	76	35.6	
Abstain from any type of sex with someone who is in or has recently traveled to an area with ZIKV	Not at all likely	126	23.6	125	24.6	34	15.4	0.004
	Somewhat likely	63	11.8	79	16.8	38	22.5	
	Likely	85	18.8	90	19.4	55	29.4	
	Very likely	220	45.8	193	39.2	66	32.7	
Use condoms with someone who is in or has recently traveled to an area with ZIKV	Not at all likely	126	23.6	126	27.4	30	19.2	0.365
	Somewhat likely	55	12.1	76	14.9	32	17.4	
	Likely	96	19.1	95	18.8	42	23.5	
	Very likely	215	45.2	187	39.0	91	40.0	

Across the three prevention behaviors related to sexual transmission—staying sober to avoid casual sex without a condom, abstaining from sex, or using a condom with someone who is in or has recently traveled to a ZIKV-affected area—more respondents from the at-risk states (45–48%) indicated they would be very likely to practice these behaviors, as compared with respondents from the other states (39–40%) and respondents from Puerto Rico (33–40%). However, differences between regions were significant only for staying sober and abstaining from sex. Significantly more respondents from Puerto Rico (32%) reported currently using a condom to prevent the possible transmission of ZIKV, as compared with respondents from the other states (20%) and the at-risk states (17%) (*P* < 0.01) (Table 5).

## DISCUSSION

This is the first study to assess and compare U.S. travelers’ ZIKV knowledge, perceived risk, self-efficacy, and behavioral intentions across three different regions: Puerto Rico, the at-risk states, and the other states. We found significant differences in knowledge, perceived risk, information seeking, and behavioral intentions across the three regions, with respondents from Puerto Rico being the most knowledgeable for nearly all types of knowledge assessed.

Respondents from Puerto Rico may have been exposed to health information campaigns regarding ZIKV that were launched in Puerto Rico in 2016, with some continuing into early 2017, given the higher rates of ZIKV in that territory.^24,36^ Public attention about emerging infections often tracks spikes in media coverage.^37^ Overall, more than 80% of respondents knew that someone could get ZIKV through a bite from an infected mosquito, and more than 60% knew that a pregnant woman could pass the virus to her fetus. These findings are fairly consistent with the findings from the two prior studies examining knowledge of these issues among Americans.^17,18^

We identified critical gaps in knowledge around sexual transmission. Less than half of respondents from the at-risk states and the other states knew that ZIKV could be transmitted sexually, as compared with three-quarters of respondents from Puerto Rico. Although respondents from Puerto Rico were more knowledgeable about sexual transmission than respondents from the at-risk states and the other states, they were less knowledgeable about ZIKV being transmitted through a blood transfusion. Given these lower levels of knowledge, future educational efforts should focus on increasing awareness about all routes of transmission but should emphasize sexual transmission and transmission through a blood transfusion.

Knowledge that ZIKV causes brain or birth defects in fetuses was generally high (80–86% correct), which is consistent with previous studies.^17,18^ However, knowledge about what people should do after visiting a ZIKV-affected area or after being exposed to ZIKV was low. Just more than half of the respondents from all three regions knew that pregnant women should be tested for ZIKV if they have visited a ZIKV-affected area. Less than one-fifth of the respondents from all three regions knew that men who had been infected should use a condom for at least 6 months after being infected, and less than 3% knew that women who may have been exposed to ZIKV should wait 2 months before trying to get pregnant. These large knowledge gaps point to the need for increased communication of accurate prevention information both before and after travel to ZIKV-affected areas.

Respondents from Puerto Rico had significantly higher risk perceptions and were significantly more concerned about getting infected with ZIKV as compared with respondents from the at-risk states and the other states. As Puerto Rico has the highest number of cases of ZIKV infection in the United States and its territories, we would expect risk perceptions to be higher among respondents from Puerto Rico.^38^ At the same time, respondents from Puerto Rico also had higher confidence in their ability to protect themselves and their families. This could be because respondents from Puerto Rico had been exposed to more detailed information about prevention of vector-borne infectious diseases, including dengue and chikungunya, which are endemic in Puerto Rico. For example, almost half of respondents from Puerto Rico had talked with a medical professional about ZIKV, as compared with about 10% of respondents from either the at-risk states or the other states. Also, significantly more respondents from Puerto Rico had actively tried to find information about ZIKV on the internet, as compared with respondents from the at-risk states and other states. Exposure to news stories or campaign messages about specific ZIKV prevention strategies also may have contributed to these differences.

The day-to-day behaviors of the Puerto Rico respondents also may have influenced both their perceived risk and their self-efficacy to prevent ZIKV because they were more likely than respondents from at-risk and other states to have already been routinely practicing ZIKV prevention behaviors; consequently, we would expect to see higher levels of self-efficacy. Their perceived risk may be higher than the perceived risk of respondents from at-risk and other states because they continued to see and hear about people from Puerto Rico becoming infected.

Knowledge about how ZIKV is transmitted also may account for the high levels of both perceived risk and self-efficacy. Because a significantly larger proportion of the Puerto Rico respondents knew that ZIKV could be transmitted sexually, they may have felt more at risk of getting infected by having sex with someone who had ZIKV and did not know it, as compared with the respondents from at-risk and other states who were less knowledgeable that ZIKV could be transmitted sexually.

This study has some limitations. First, the study only included respondents who previously had heard about ZIKV and indicated they planned to travel to a ZIKV-affected area in 2017. Second, the respondents from Puerto Rico were recruited using a different method than the respondents from the at-risk states and the other states. Consequently, the sample of respondents from Puerto Rico may not be representative of the Puerto Rican population. Third, although differences between the three regions may be suggestive of different levels of exposure to information about ZIKV, this study did not measure these differences. Consequently, the findings can only describe differences and cannot attribute differences to specific communication or education initiatives.

Overall, the findings suggest that significant gaps in U.S. travelers’ knowledge exist in terms of how to prevent ZIKV transmission during travel to an area that has had reported cases of ZIKV transmission via mosquitoes and through sex with a partner who has been infected. These lower levels of accurate knowledge about sexual transmission are especially concerning. Consequently, opportunities exist for health care providers, public health officials, and the travel industry to advise travelers about the risk of ZIKV and prevention methods, particularly condom use and delaying pregnancy for specified period after travel to a ZIKV-affected area. Specifically, health care providers, should ask both male and female patients about both pregnancy and travel plans, and discuss how to prevent ZIKV with those who are traveling to or living in ZIKV-affected areas. For patients who are sexually active and do not intend to become pregnant, health care providers should make sure that patients have and know how to use a reliable contraceptive method. For patients who do plan to conceive, health care providers should follow CDC’s advice for counseling patients about pregnancy if they are in or traveling to a ZIKV-affected area. Clear communication from health care providers will be crucial because they remain the most trusted source of health information.^39,40^
